# A new crystalline daidzein-piperazine salt with enhanced solubility, permeability, and bioavailability

**DOI:** 10.3389/fphar.2024.1385637

**Published:** 2024-07-22

**Authors:** Jiacheng Meng, Chenxu Qiu, Chenyue Lu, Xin He, Xinghua Zhao

**Affiliations:** College of Veterinary Medicine, Hebei Agricultural University, Baoding, China

**Keywords:** daidzein, piperazine, cocrystallization, dissolution, permeability, bioavailability

## Abstract

To overcome the poor solubility, permeability, and bioavailability of the plant isoflavone daidzein (DAI), a novel salt of DAI with anhydrous piperazine (PIP) was obtained based on cocrystallization strategy. The new salt DAI-PIP was characterized by powder X-ray diffraction (PXRD), differential scanning calorimetry (DSC), thermogravimetric analysis (TGA), Fourier-transform infrared (FT-IR) spectroscopy, and optical microscopy. The results showed that the maximum apparent solubility (S_max_) of DAI-PIP increased by 7.27-fold and 1000-fold compared to DAI in pH 6.8 buffer and water, respectively. The peak apparent permeability coefficient (P_*app*_) of DAI-PIP in the Caco-2 cell model was 30.57 ± 1.08 × 10^−6^ cm/s, which was 34.08% higher than that of DAI. Additionally, compared to DAI, the maximum plasma concentration (C_max_) value of DAI-PIP in beagle dogs was approximately 4.3 times higher, and the area under the concentration-time curve (AUC_0-24_) was approximately 2.4 times higher. This study provides a new strategy to enhance the dissolution performance and bioavailability of flavonoid drugs, laying a foundation for expanding their clinical applications.

## 1 Introduction

**FIGURE 1:**
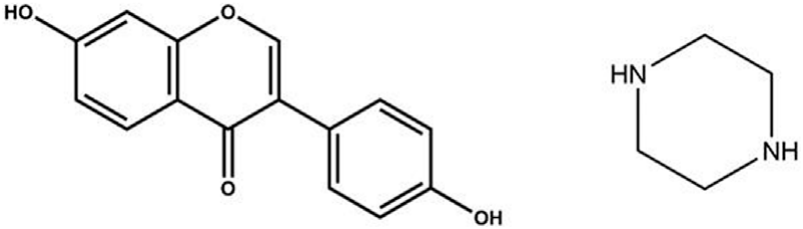
Chemical Structures of Daidzein (DAI, MW = 254.24 g/mol, *pK*_a_ = 7.43) and Piperazine (PIP, MW = 86.14 g/mol, *pK*_a_ = 5.49).

Daidzein (DAI; 4′,7-dihydroxyisoflavone) (Figure 1A) is a naturally occurring phytoestrogen (Alshehri et al., 2021) primarily obtained from leguminous plants. It is classified as a phytoestrogen because of its structural and functional similarities with the primary female sex hormone, 17-β-estradiol (E2) (Cederroth and Nef, 2009). Its estrogenic effects are useful in treating breast cancer (Sathyamoorthy and Wang, 1997), prostate cancer (Adjakly et al., 2013), thyroid carcinoma (Vivacqua et al., 2006), osteoporosis (Vitale et al., 2013), and menopausal symptoms. Additionally, DAI has been found to exert other pharmacological effects, such as scavenging free radicals and preventing oxidative damage to the body, and reducing serum total cholesterol, low-density lipoprotein cholesterol, and triacylglycerol levels (Yuri et al., 2013; Kitamura et al., 2020). It also inhibits inflammatory response and production of cytokines (Ginwala et al., 2019; Jucá et al., 2020), promotes the production of nitric oxide by vascular endothelial cells, dilates blood vessels, and benefits the cardiovascular system (Laddha and Kulkarni, 2021). Nevertheless, due to the formation of a planar structure by the double bond between the 2nd and 3rd carbon atoms of DAI and the tight molecular arrangement, solvent molecules cannot easily penetrate the molecular structure. DAI has been identified as a class IV drug in the Biopharmaceutics Classification System (BCS) owing to its low water solubility (0.31 μg/mL) (Nan et al., 2014; Panizzon et al., 2019) and poor permeability (Waldmann et al., 2012). These physicochemical characteristics limit its bioavailability and biological effects (Pandey et al., 2024).

Cocrystallization is an efficient method widely utilized for improving the physicochemical characteristics of active pharmaceutical ingredients (APIs) (Cerreia Vioglio et al., 2017; Kumar et al., 2018) and enhancing drug bioavailability (Emami et al., 2018) by creating multicomponent crystalline solids. Pharmaceutical cocrystallization technology involves the formation of a new type of drug molecule via hydrogen bonding, π-π conjugation, or other noncovalent-bond formation between APIs and cocrystal former (CCF) in a stoichiometric ratio. The use of crystal engineering in the pharmaceutical industry has become increasingly prevalent in recent years to enhance the efficacy of APIs (Duggirala et al., 2016). Several strategies have been reported to enhance the solubility and bioavailability of DAI. For example, DAI-loaded solid lipid nanoparticles (SLNs) with PEGylated phospholipid as stabilizer were successfully prepared by hot homogenization method. Pharmacokinetic analysis showed that the DAI-loaded SLNs could significantly increase circulation time compared with orally administrated DAI suspension or intravenously delivered DAI solution (Gao et al., 2008). Self-microemulsifying drug delivery system (SMEDDS) composed of oil, surfactant and cosurfactant was prepared for oral administration of DAI, which increased the relative bioavailability by 2.5 times (Shen et al., 2010). However, these preparation processes involve complex procedures and the addition of various carrier materials. In contrast, the preparation of cocrystals is simpler. Moreover, the mechanochemistry method used for cocrystal preparation is regarded as environmentally friendly, because it can be conducted without solvents or with only trace/catalytic amounts of solvent, unlike traditional solution reactions that often use large volumes of solvents (Solares-Briones et al., 2021). Currently, five types of DAI cocrystals have been prepared. Bhalla et al. (2019) prepared three cocrystals (DAI-isonicotinamide, DAI-cytosine, and DAI-theobromine) that led to a nearly two-fold increase in the solubility of DAI in water and a 2.12-fold increase in C_max_ in rat plasma. The other two cocrystals, namely, DAI-theophylline cocrystal and DAI-4,4-bipyridine cocrystal (Bolus et al., 2020), increased the dissolution of DAI by 2.03 and 2.27 times, respectively. However, the aforementioned studies did not include investigations into permeability. Therefore, the characterization of DAI cocrystals is incomplete, and their enhancement effect on the water solubility of DAI is limited.

Piperazine (PIP) is a six-membered heterocycle containing two nitrogen atoms arranged diagonally (Figure 1B) (Staack, 2007; Patel and Park, 2013). PIP plays a crucial role in balancing the pharmacokinetic profile of designed compounds and has been widely used in developing various pharmacologically active compounds, including anthelmintics (Shaquiquzzaman et al., 2015), antioxidants (Berczyński et al., 2020), antibacterials (Girase et al., 2021), anti-inflammatory agents (Sharma S. et al., 2020), antidiabetics (Devine et al., 2020), and antidepressants (Li et al., 2009). In this study, PIP was chosen as the cocrystal former (CCF) for three reasons. First, structurally, PIP has two amino groups that can act as proton acceptors, while most flavonoids contain a large number of phenolic hydroxyl groups and carbonyl groups, which act as proton donors and acceptors. This also facilitates the interaction of PIP with DAI to form new multicomponent solid forms. Second, functionally, PIP exhibits excellent water solubility as a small molecule (1,000 mg/mL in water) (Roy et al., 2023). It is found to be effective in enhancing the solubility and dissolution-rate of various drugs such as 5-fluorouracil (Muresan-Pop et., 2020) and sulfamethazine (Wang et al., 2021). Finally, PIP derivatives are penetration enhancers that can improve drug absorption by modulating epithelial structure and reducing transepithelial resistance (Zheng et al., 2020), which may also enhance DAI absorption in the intestinal tract.

This work focused on a new solid state salt of DAI and PIP, aiming to improve the solubility, permeability, and pharmacokinetic behavior of DAI. The newly obtained solid form of DAI was prepared by liquid-assisted grinding (LAG) method and slurry method. It was then systematically characterized by single crystal X-ray diffraction, thermal analysis, and Fourier transform infrared spectroscopy. Furthermore, its physicochemical properties including dissolution behavior, permeability, as well as pharmacokinetics were evaluated.

## 2 Materials

DAI (purity ≥98%) was purchased from Purify Technology Development Co., Ltd. (Chengdu, China). Anhydrous PIP (purity ≥99%) was purchased from Aladdin Biochemical Technology Co., Ltd. (Shanghai, China). DAI standard (purity ≥99%) was purchased from Solarbio Technology Co., Ltd. (Beijing, China). Minimum Eagle’s medium (MEM) (including nonessential amino acids) was purchased from Punuosai Biotechnology Co., Ltd. (Wuhan, China). HPLC-grade methanol was purchased from Thermo Fisher Scientific (Shanghai, China). All other chemicals and solvents were of analytical reagent grade.

## 3 Methods

### 3.1 Synthesis of DAI-PIP salt and single crystals

DAI-PIP salt was prepared using LAG method (Xu et al., 2012) and slurry method (Wang et al., 2022) at ambient temperature. The LAG method was performed by mixing stoichiometric amounts of DAI (50.848 mg, 0.2 mmol) and PIP (17.228 mg, 0.2 mmol) with 20 μL methanol in a 2-mL Eppendorf tube. The mixture was ground with a stainless steel milling ball (5 mm in diameter) for 30 min at a frequency of 20 Hz on a vibration apparatus (GT300, Beijing Grinder Instrument Co., Ltd., China).

In the slurry method, DAI (50.85 mg, 0.2 mmol) and PIP (17.23 mg, 0.2 mmol) were suspended in 50 mL methanol and stirred at 250 rpm using a magnetic stirring bar. The solid was filtered after 24 h and dried at ambient temperature for 24 h.

To obtain single crystals, DAI (50.84 mg, 0.2 mmol) and PIP (17.2 mg, 0.2 mmol) in a 1:1 ratio were dissolved in 6 mL methanol and evaporated slowly in a fume hood at room temperature. After 5–7 days, colorless cluster-shaped single crystals of DAI-PIP were harvested.

### 3.2 Single-crystal X-ray diffraction (SXRD)

X-ray diffraction of single crystal was performed at 150 K using an Agilent Technologies Gemini A diffractometer (Rigaku, Japan) with Mo-Kα radiation (λ = 0.71073 Å). Cell refinement and data reduction were performed using the CrysAlisPRO program (Li J. et al., 2021). The crystal structure was solved by direct methods and refined using the full-matrix least-squares method on F2 with SHELXTL program in the Olex-2 software. During refinement, anisotropic displacement parameters were assigned to all nonhydrogen atoms, while ideal positions with fixed isotropic thermal parameters were assigned to all hydrogen atoms. The resulting crystal structure was drawn using Mercury 4.3.1.

### 3.3 Powder X-ray diffraction (PXRD)

Powder samples were analyzed using an X-ray diffractometer (SmartLab Studio II, Rigaku, Japan) equipped with a Cu Kα radiation (λ = 1.54178 Å) detector. The tube voltage and amperage were 15 mA and 40 kV, respectively. All samples were analyzed at room temperature from 3° to 35° (2θ, step size was 0.02° and dwell time at each step was 0.1 s).

### 3.4 Differential scanning calorimetry (DSC) and thermogravimetric (TG) analysis

DSC thermograms were obtained using a DSC 3 calorimeter (METTLER TOLEDO, Switzerland). Samples (3–5 mg) were weighed into DSC sample pans, which were hermetically sealed and Li et al., 2021 d-pierced. Then, they were measured in the temperature range of 35°C–340°C with a heating rate of 10°C/min under nitrogen purge at a flow rate of 50 mL/min. TG analysis was performed using a TG-209 F3 instrument (Netzsch, Germany). Approximately 5–10 mg of sample was placed in open aluminum oxide pans and heated at a heating rate of 10°C/min to 340°C under nitrogen atmosphere.

### 3.5 Fourier-transform infrared (FT-IR) spectroscopy

FT-IR spectroscopy was performed using a Bruker Alpha FT-IR spectrometer (Ettlingen, Germany). Powder samples were mixed with KBr at a 1:100 ratio, and pressed into a pellet using a hydraulic press at a pressure of 10 MPa for 60 s. Data were collected for all samples from 4,000 to 400 cm^−1^, with a resolution of 0.1 cm^−1^ (Li X. et al., 2021).

### 3.6 Optical microscopy

The surface morphology of DAI, PIP, and DAI-PIP was observed using an optical microscope (Cx31, Olympus Corporation, Japan). Approximately 5–10 mg of the sample was placed on a glass slide to acquire optical microscopic images magnified by 100 times.

### 3.7 High-performance liquid chromatography (HPLC)

A Model 1,525 system (Waters Corporation, Milford MA, United States) equipped with a photodiode array (PDA) detector (Waters 2998, Milford MA, United States) and a SunFire C18 column (4.6 mm × 250 mm, 5 μm, Waters, Ireland) was used for HPLC analysis. The column temperature was maintained at 37°C and the autosampler temperature was room temperature. The mobile phase consisted of methanol and H_2_O (70:30, *v/v*) at a flow rate of 1.0 mL/min. The detection wavelength of the UV detector was 248 nm and the injection volume of sample was 20 µL. The standard curve ranged from 10 μg/mL to 0.078125 μg/mL.

### 3.8 Powder dissolution experiments

The powder dissolution profiles of DAI and DAI-PIP were obtained using a dissolution device (D-800LS, Precision Instrument Factory of Tianjin University, Tianjin, China). The powders were sieved using standard sieves to control particle size within 75–150 µm. During the powder dissolution, excess powder was suspended in 900 mL of pH 6.8 phosphate buffer in a water-jacketed beaker and stirred at 250 rpm, 37°C. At predetermined time points (5, 10, 15, 20, 30, 45, 60, 90, 120, and 240 min), an aliquot of the slurry was withdrawn, and immediately filtered through a 0.22-µm polyether sulfone filter membrane. The filtrates were diluted to an appropriate concentration, and analyzed using HPLC.

### 3.9 *In vitro* Caco-2 cell monolayer transport analysis

Caco-2 cells were obtained from Punuosai Biotechnology Co., Ltd. (Wuhan, China). The cells were cultured in MEM supplemented with 20% fetal bovine serum (FBS), 1% non-essential amino acids, 1% penicillin, and 1% streptomycin in an atmosphere of 5% CO_2_ and 100% relative humidity at 37°C. All cells used in this experiment were between passages 35 and 40.

The cytotoxicity of DAI, PIP, and DAI-PIP was evaluated by Cell Counting Kit-8 (CCK-8) assay (Song et al., 2023). A cell suspension with a density of 1 × 10^5^ cells/mL was inoculated onto a 96-well plate. The cells were treated with various concentrations of DAI (10, 20, 30, 40, 50, and 60 μg/mL) for 24 h. After the cells were washed with phosphate-buffered saline solution (pH 7.4), 10 μL CCK-8 solution (10%) was added to each well. Then, the cells were incubated in the dark for 2 h before measuring the optical density (OD) at 450 nm using a microplate reader (K3, LabServ, Shanghai). The percent cell viability (%) at different drug concentrations was calculated from Eq. 1, as shown below:

$$\text{Cell viability }\left(\%\right)=\left({\text{OD}}_{\text{test}}\;–\;{\text{OD}}_{\text{blank}}\;/\;{\text{OD}}_{\text{control}}\;–\;{\text{OD}}_{\text{blank}}\right)\times 100\%$$ (1)

The cell viabilities of PIP and DAI-PIP were evaluated using the same method according to the principle of equivalent amount of DAI.

Caco-2 cells were seeded on 12-well Transwell^®^ (Corning Costar, Lowell, MA, United States) culture plates (pore size 0.4 µm, surface area 1.12 cm^2^) at 5 × 10^4^ cells/mL. On the 3rd, 5th, 7th, 13th, 18th, and 21st days of culture, the transepithelial electrical resistance (TEER) value was measured using an EVOM2 transmembrane resistance meter (Millipore, United States) to evaluate the integrity of the Caco-2 cell monolayer.

The efflux function of the cell model was evaluated using Rhodamine 123. For the experiment from AP to BL side: 0.5 mL of Rhodamine 123 solution was added to the AP side and 1.5 mL of blank HBSS solution was added to the BL side. For the experiment from BL to AP side: 1.5 mL of Rhodamine 123 solution was added to the BL side and 0.5 mL of blank HBSS solution was added to the AP side. Samples were collected at 30, 60, 90, and 120 min, and 200 μL of each sample was used to measure the optical density (OD) value. Permeability coefficient (P_*app*_) and Efflux ratio (ER) was calculated from Eq. 2 (Wang et al., 2017) and the permeability coefficient (Papp) was calculated from Eq. 3 for each direction in triplicate.

$$\text{ER}=P_{app}\;\left(\text{BL}\to \text{AP}\right)\;/\;P_{app}\;\left(\text{AP}\to \text{BL}\right)$$ (2)

After washing the Caco-2 cell monolayer twice with prewarmed Hank’s Balanced Salt Solution (HBSS), the transport experiments were performed by adding the final concentration of DAI (0.5 mL) to the apical side (AP) as the donor chamber, and adding HBSS (1.5 mL) to the basolateral side (BL) as the receiving chamber. The plate was incubated at 37°C and 200 μL of sample was collected from the BL side at 30, 45, 60, 90, and 120 min. After the withdrawal of samples, an equal volume of fresh HBSS was added to the BL side immediately (Yang et al., 2014). The samples were sealed with parafilm and stored at −20°C until HPLC analysis. All experiments were performed in triplicate.

The apparent permeability coefficient (P_*app*_) for DAI was calculated using Eq. 3 (Ndayishimiye et al., 2022).

$$P_{app}=\frac{\text{dQ}}{\text{dt}}*\frac{1}{A*C_{0}}\;\left(\text{cm}/s\right)$$ (3)

where Q refers to the cumulative transport amount, i.e., the total drug quantity on the receiving side (μg); dQ/dt represents the transport rate of the drug through the Caco-2 cell monolayer (μg/s); A denotes the chamber membrane area (1.12 cm^2^); and C_0_ is the initial concentration of DAI on the AP side (μg/mL).

### 3.10 *In vivo* pharmacokinetic studies in beagle dogs

Pharmacokinetic analysis was carried out in male Beagle dogs (12–16 kg, Masi Biotechnology Co., Ltd., Beijing, China). After overnight fasting, six healthy dogs were chosen for the study and randomly divided into two groups before dosing (n = 3 in each group). All animal experiments in this study were approved by the Institutional Animal Ethics Committee of Hebei Agricultural University (No.2022161).

Sieved powder samples (75–150 µm) of DAI and DAI-PIP salt were suspended in 0.5% sodium carboxymethylcellulose and administered orally at a single dose of 40 mg/kg. Blood samples (about 1,000 μL) were collected from the cephalic vein of the forelimbs at 5, 15, 30, 45, 60, 120, 240, 360, 540, and 720 min after administration. Normal heparin was used as anticoagulant. The blood samples were centrifuged at 6,000 rpm for 10 min and the plasma samples were stored at −20°C until HPLC analysis. For the analysis, 100 μL of each plasma sample was added to a 5 mL glass tube followed by the addition of 200 μL acetonitrile. The mixture was vortexed for 120 s and then centrifuged for 10 min at 12,000 rpm to precipitate proteins (Wang et al., 2022). The supernatant was passed through a membrane filter prior to HPLC analysis. Pharmacokinetic parameters, such as maximum plasma concentration (C_max_), time required to reach C_max_ (T_max_), area under the plasma concentration–time curve (AUC_0-24_), and half-life (t_1/2_) were calculated with a noncompartmental model using DAS 2.0.

### 3.11 Statistical analysis

Data were presented as mean ± standard error of the mean and analyzed using SPSS 20.0 (SPSS Inc., Chicago, IL, United States). Statistical significance was determined using one-way analysis of variance (ANOVA), with *p* < 0.05 and *p* < 0.01 considered to be statistically significant.

## 4 Results and discussion

### 4.1 Single-crystal X-ray diffraction

**FIGURE 2:**
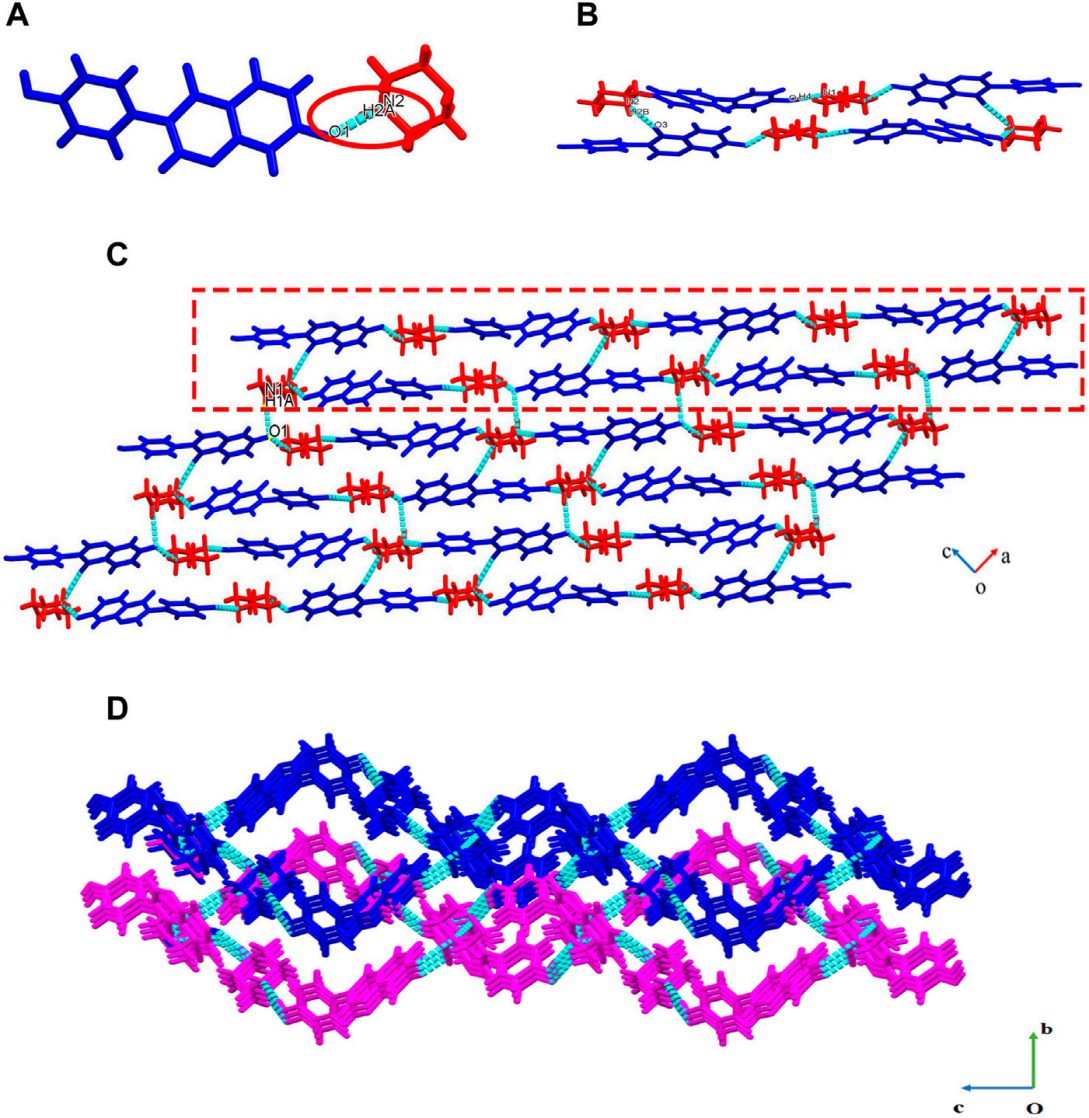
Crystal structure of DAI-PIP **(A)** asymmetric unit **(B)** octamer **(C)** 2D net-like structure, 1D chain (within the red box) **(D)** 3D packing structure view into a-axis.

DAI-PIP crystallizes in a triclinic space group P21/c (CCDC deposition number 2180471), with one DAI anion and one PIP cation in the asymmetric unit (Figure 2A), within which the H2A atom is transferred from DAI to PIP. It is generally accepted that salts are formed when the Δ*pK*_a_ (Δ*pK*_a_ = *pK*_a_ of base—*pK*_a_ of acid) values are greater than 3, and cocrystals are formed when the Δ*pK*_a_ values are negative. Either a cocrystal or a salt will form when the Δ*pK*_a_ is between 0 and 3 (Childs et al., 2007). The *pK*_a_ values of DAI and PIP are 7.43 and 5.49, respectively, and their Δ*pK*_a_ is between 0 and 3. Hence, they will form either salt or cocrystal according to the rules. Indeed, the reaction of DAI with PIP gave a new salt of DAI-PIP.

In the asymmetric unit, one DAI anion interacts with one PIP cation through N2-H2A…O1 (2.549 Å) hydrogen bonds (Figure 2A). The four adjacent asymmetric units are connected to form an octamer structure through O4-H4…N1 (2.710 Å) and N2-H2B…O3 (2.841Å) (Figure 2B). One-dimensional chains are formed by connecting adjacent tetramer structures through O4-H4…N1 (2.710 Å) hydrogen bonds (rectangular annotation in Figure 2C). Two-dimensional structures are formed between one-dimensional chains along the a-axis through N1-H1A… O1 (3.021 Å) hydrogen bonds (Figure 2C). Lastly, three-dimensional structures are formed between two layers of two-dimensional planes along the b-axis through short contact action, van der Waals forces, etc. (Figure 2D). Crystallographic data and key refinement parameters for the structure of DAI-PIP are listed in Table 1, and information on selected hydrogen bonds is provided in Table 2.

**TABLE 1:** Crystallographic data and refinement parameters for DAI-PIP.

Formula		DAI-PIP	
Formula weight		340.37	
Temperature (K)		114.05 (10)	
Crystal system		monoclinic	
Space group		P2_1_/c	
a (Å)		12.117 (2)	
b (Å)		6.3353 (8)	
c (Å)		21.058 (4)	
α (deg)		90.00	
β (deg)		95.526 (16)	
γ (deg)		90.00	
volume (Å3)		1,609.0 (5)	
Z		4	
Calculated density (g/cm^3^)		1.405	
GOF on F^2^		0.936	
*R*_int_		0.1469	
*R*_1_ [*I* ≥ 2σ(I)]^a^		0.0843, 0.1030	
w*R*_2_		0.2118, 0.1499	
CCDC No.		2180471	

**TABLE 2:** Hydrogen bonding distance and angles for DAI-PIP

	D-H…A	D-H(Å)	H…A(Å)	D…A(Å)	<D-H…A (°)
DAI-PIP	O4-H4…N1	0.820	1.896	2.710	172.30
	N1-H1A…O1	0.860	2.184	3.021	164.08
	N2-H2A…O1	0.900	1.669	2.549	164.88
	N2-H2B…O3	0.900	2.098	2.841	139.15

Symmetry codes: (i) -x, -y+2, -z+2 (ii) -x+5/2, y+1/2, -z+1/2 (iii) -x+2, -y+1, z.

### 4.2 Powder X-ray diffraction

**FIGURE 3:**
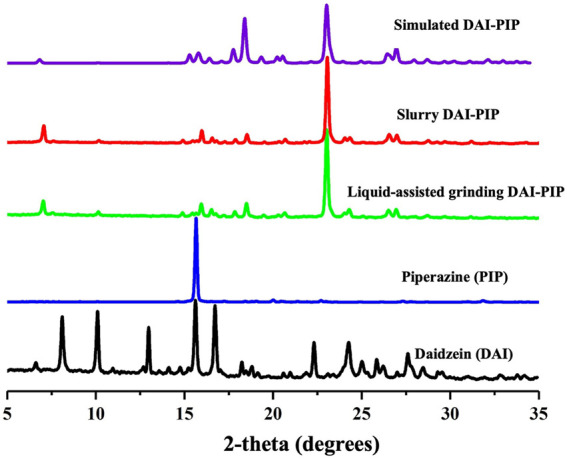
PXRD patterns of DAI, PIP, DAI-PIP, and simulated DAI-PIP.

PXRD is a reliable technique for identifying the formation of new crystal phases (Hiendrawan et al., 2016; Sanphui et al., 2011). In the PXRD patterns of the grinded powder and the slurry powder, the characteristic peaks of DAI (6.53, 8.07, 12.9, and 22.4°) and PIP (20.0, and 21.4°) disappeared, and some new peaks (7.06, 16.0, 23.1, and 26.4°) appeared (Figure 3). These results suggested that the powder was not a simple physical mixture between API and CCF, and that a new solid phase was formed. Furthermore, the PXRD pattern of the bulk powder was in good accordance with the simulated pattern generated from single crystal diffraction data, indicating the generation of highly pure phases.

### 4.3 Thermal analysis

**FIGURE 4:**
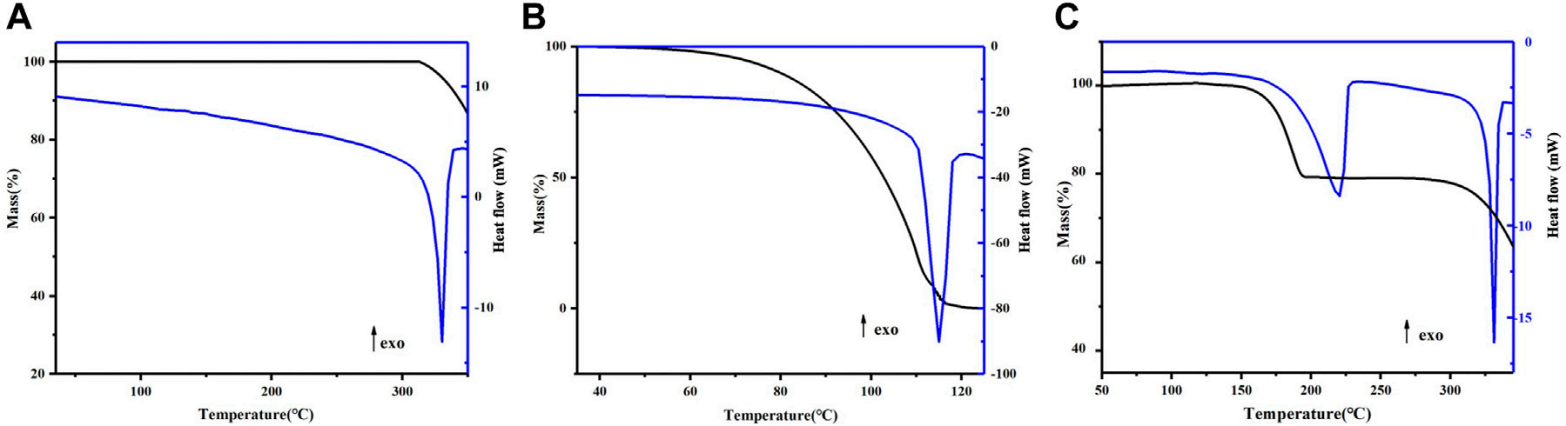
DSC and TGA curves of DAI **(A)**, PIP **(B)**, DAI-PIP **(C)**.

The thermal behavior of DAI-PIP was investigated by DSC and TG, and the results are presented in Figure 4C. DAI showed an endothermic peak at 330.1°C (Figure 4A), consistent with the reported melting temperature of DAI. The melting point of PIP is 115.1°C (Figure 4B), above which decomposition begins.

TG curve of DAI-PIP showed a weight loss of 23.6% corresponding to the loss of PIP molecule (theoretical weight percentage: 25.9%). Then, upon further heating, only DAI remained, indicating that the sample was pure DAI after the complete loss of PIP. This finding was also consistent with results derived from the DSC thermogram. The first endotherm can be attributed to the dissociation and release of PIP, whereas the second endotherm corresponded to the melting of the remaining DAI.

### 4.4 FT-IR spectroscopy

**FIGURE 5:**
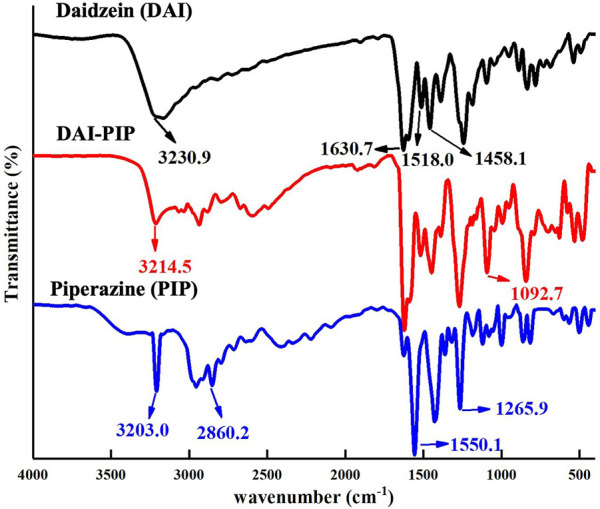
FT-IR spectra of DAI, PIP, and DAI-PIP.

The hydrogen bonding patterns of a molecule change after the formation of a salt or cocrystal, which in turn changes the vibrational modes associated with the functional groups and their corresponding infrared frequencies. The FT-IR spectrum of DAI shows free O−H stretching absorption peak at 3,230.9 cm^−1^, C=C stretching absorption peaks of benzene ring at 1,458.1 cm^−1^, 1,518 cm^−1^, and 1,630.7 cm^−1^, and C=O stretching absorption peak at 1,630.7 cm^−1^ (Bhalla et al., 2019). The N−H and C−H stretching of PIP showed characteristic peaks at 3,023.0, 1,550, and 2860.2 cm^−1^, while the C–N stretching vibration peak was located at 1,265.9 cm^−1^ (Figure 5). Hydrogen bond formation makes the absorption peaks of DPI-PIP more complex. The O−H stretching vibration peak of DAI shifted from 3,230.9 cm⁻^1^–3,214.5 cm⁻^1^, and the C−N stretching vibration peak of PIP shifted from 1,265.9 cm⁻^1^–1,092.7 cm⁻^1^. This shift can be attributed to the formation of hydrogen bonds (O−H···N) between DAI and PIP.

### 4.5 Optical microscopy

**FIGURE 6:**
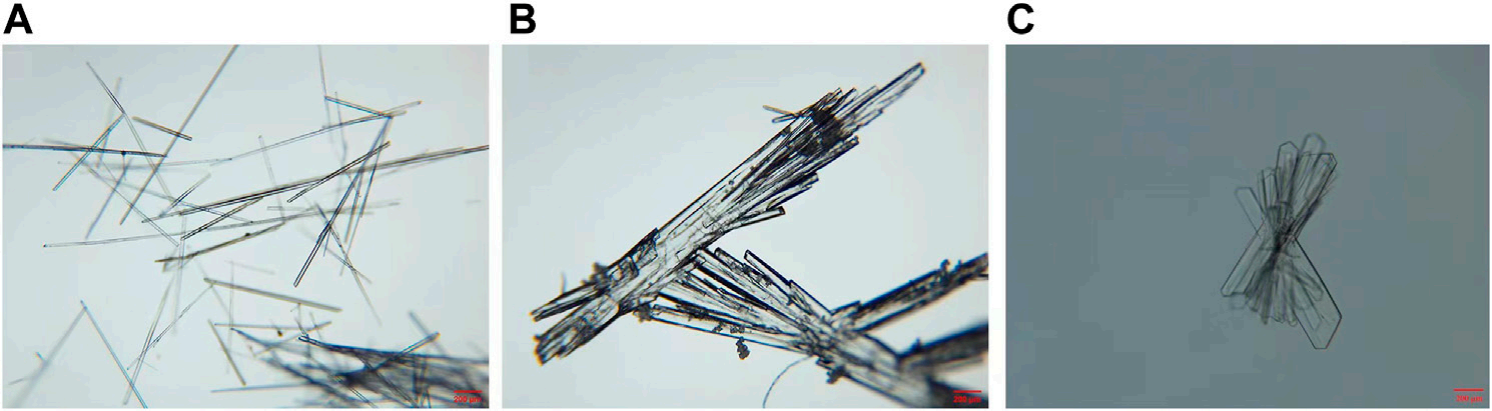
Optical microscopy images of **(A)** DAI, **(B)** PIP, and **(C)** DAI-PIP.

Micrographs of DAI, PIP, and DAI-PIP are shown in Figure 6. DAI-PIP salt appeared as transparent cluster crystals, distinct from DAI shaped as acicular crystals and PIP which appeared as columnar crystals.

### 4.6 Powder dissolution

The dissolution rate of API is of paramount importance in pharmaceutical development, and higher dissolution rate may result in better absorption and greater solubility-limited bioavailability (Pang et al., 2023).

**FIGURE 7:**
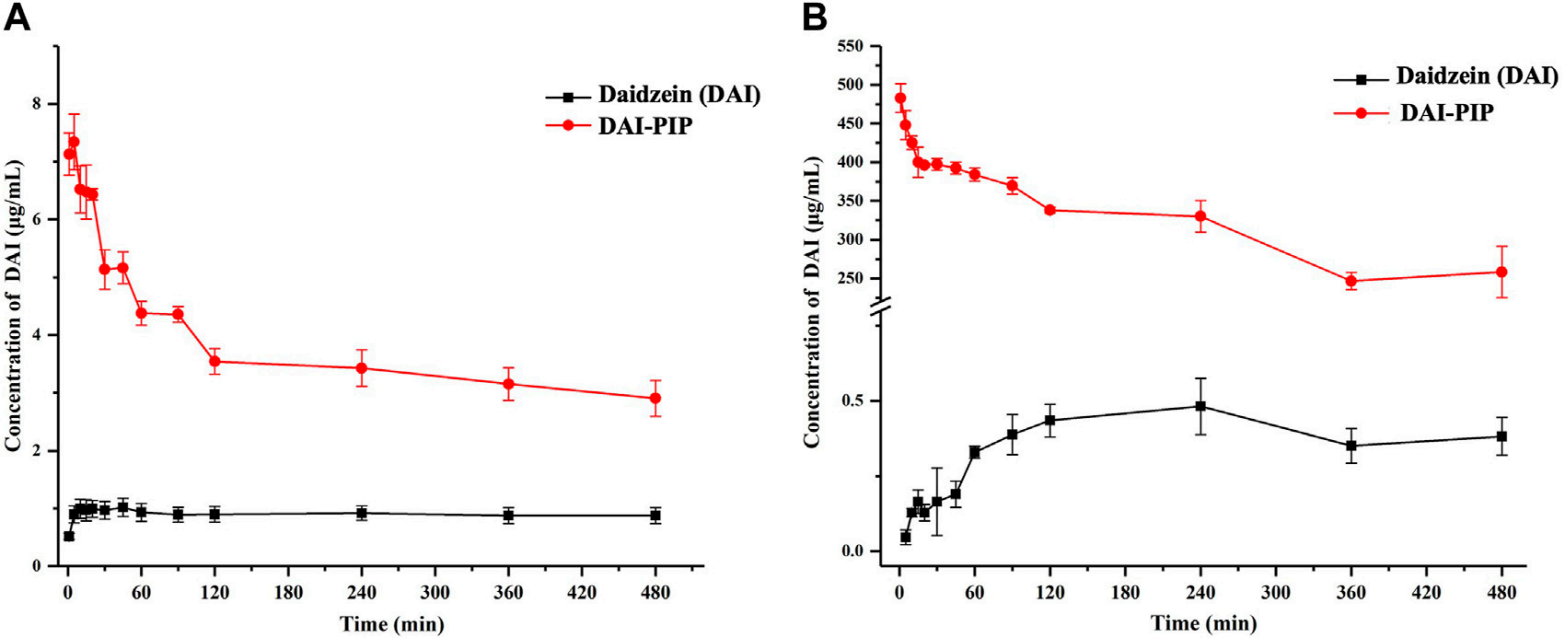
Powder dissolution profiles of DAI and DAI-PIP in pH 6.8 buffer media **(A)** and water **(B)**.

The powder dissolution results of DAI and DAI-PIP in water and pH 6.8 PBS buffer are shown in Figure 7A. The maximum apparent solubility (S_max_) values of DAI and DAI-PIP in pH 6.8 buffer were 1.01 ± 0.06 and 7.34 ± 0.48 μg/mL, with the peak times of 45 and 5 min, respectively. The apparent solubility of DAI-PIP exhibited a short-term improvement and its S_max_ value was 7.27 times higher than that of pure DAI.

However, the S_max_ values of DAI and DAI-PIP in water were considerably higher than those in pH 6.8 buffer (Figure 7B). The S_max_ of DAI reached 0.48 ± 0.09 μg/mL within 240 min and maintained a plateau. Interestingly, the S_max_ of DAI-PIP was reached rapidly, which was about 1000-fold (483.00 ± 18.60 μg/mL) higher than that of pure DAI. Moreover, the apparent solubility of DAI-PIP was still 680-fold (258.46 ± 12.99 μg/mL) higher than that of DAI at the end of the experiment (480 min).

The difference in dissolution behavior in water and pH 6.8 buffer was due to the basic nature of PIP which influenced the ionization of DAI through the change in environmental pH. As shown in Table 3, in both water and pH 6.8 buffer, the environmental pH values of DAI-PIP were 7.54 ± 0.02 and 9.76 ± 0.03, respectively, which were higher than those of pure DAI. In addition, the variation of pH values in pH 6.8 buffer was very small due to the buffer capacity of the PBS solution.

**TABLE 3:** Environmental pH Values for DAI and DAI−PIP.

Medium	pH	
	DAI	DAI-PIP
pH6.8 buffer	6.74 ± 0.03	7.54 ± 0.02
Water (pH 7.0)	3.80 ± 0.06	9.76 ± 0.03

The higher apparent solubility of DAI-PIP in water was caused by two phenolic hydroxyl groups in the structure of DAI, which are acidic and easily soluble in alkaline solvents.

**FIGURE 8:**
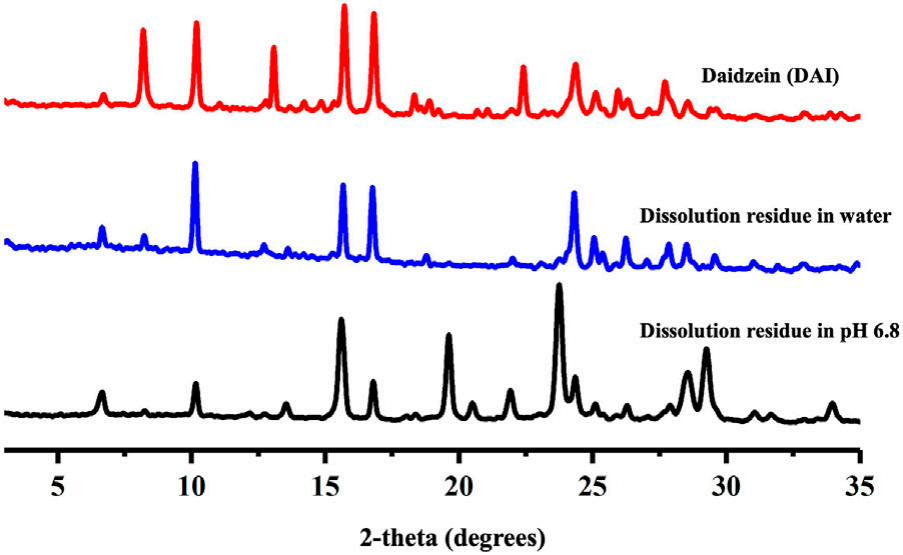
PXRD of DAI-PIP after dissolution.

After reaching the peak, the DAI concentration of DAI-PIP in both water and pH 6.8 decreased slowly over time. This is a common “spring and parachute” effect reported in many cocrystal and salt systems (Xu et al., 2012). The above result indicated that the salt underwent phase transition, which was further confirmed by PXRD tests of the undissolved solids (Figure 8). This phenomenon was caused by the large solubility difference between the API and CCF (Dai et al., 2020). The solubility behavior of cocrystal/salt systems is typically correlated with the solubility of the included guest component (Ren et al., 2022). In this study, the guest component, PIP, exhibits excellent water solubility, with a value of 1,000 mg/mL (25°C in water) (Roy et al., 2023). When DAI-PIP was added into the solvent, the crystal structure of the salt was easily disrupted due to the stronger interaction between PIP and the solvent. Rapidly dissociated APIs form supramolecular aggregates or clusters from randomly oriented molecules, similar to amorphous drug phases. This also contributes to the dispersion of APIs in the dissolution medium, thus improving drug solubility (Babu and Nangia, 2011). Over time, the amorphous drug phases convert to a stable crystalline state. The higher aqueous solubility of DAI in the first stage may lead to better absorption and bioavailability *in vivo*.

### 4.7 Permeability studies

**FIGURE 9:**
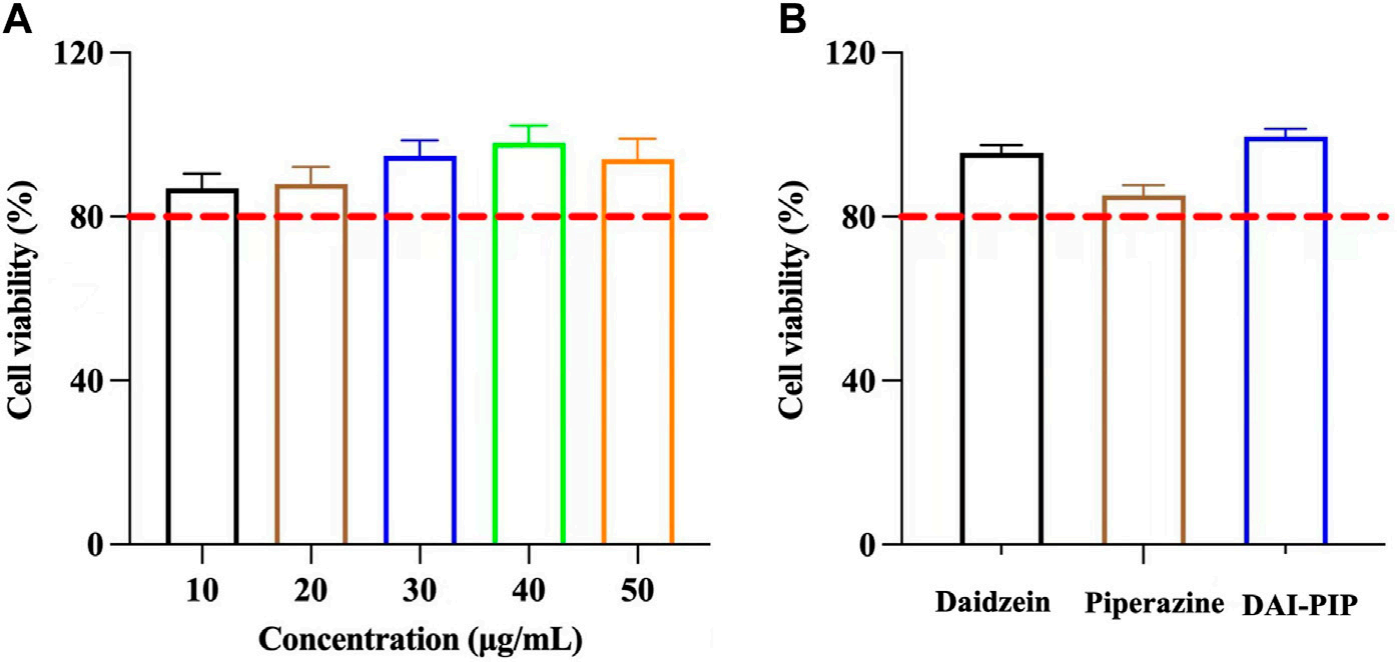
**(A)** Cell viability of DAI under different concentrations **(B)** Cell viability of PIP and DAI-PIP with the concentration of DAI (50 μg/mL) and PIP (16.94 μg/mL).

Viability of Caco-2 cells was used to evaluate the cytotoxicity of DAI and DAI-PIP. Generally, cell viability higher than 80% indicates that the compound is nontoxic to cells (Sharma M. et al., 2020). The results of cell viability are shown in Figure 9A. The cell viability values at five different concentrations were 86.95% ± 3.58%, 88.1% ± 4.05%, 94.72% ± 3.8%, 98.02% ± 4.28%, and 94.02% ± 5.04%, respectively. The results showed that DAI possessed a dose-dependent inhibitory effect on the Caco-2 cells. A concentration of 50 μg/mL DAI was selected for subsequent experiments. The PIP concentration was calculated based on the molar ratio of the new salt, and the test concentration of PIP was 16.94 μg/mL. The cell viability values of PIP and DAI-PIP were 85.25% ± 4.67%, and 99.60% ± 4.76%, respectively (Figure 9B). The above results showed the safety of DAI, PIP and DAI-PIP at the selected concentration.

The TEER value serves as a vital biophysical indicator to confirm the integrity of the Caco-2 cell monolayer. A TEER value greater than 500 indicates that the cells are tightly connected (Henri et al., 2021). The TEER value increased gradually and reached over 800 Ω cm^2^ in 21 days. The ER value of the efflux marker Rhodamine 123 was 4.76 (Table 4), indicating that the efflux function of the cellular monolayer was suitable for subsequent experiments.

**TABLE 4:** The transport results of sodium fluorescein.

Analyte	P_*app*_/×10^–6^ cm·s^-1^120 min		ER
	AP-BL	BL-AP	
Rhodamine 123	0.179 ± 0.061	0.852 ± 0.016	4.76

**FIGURE 10:**
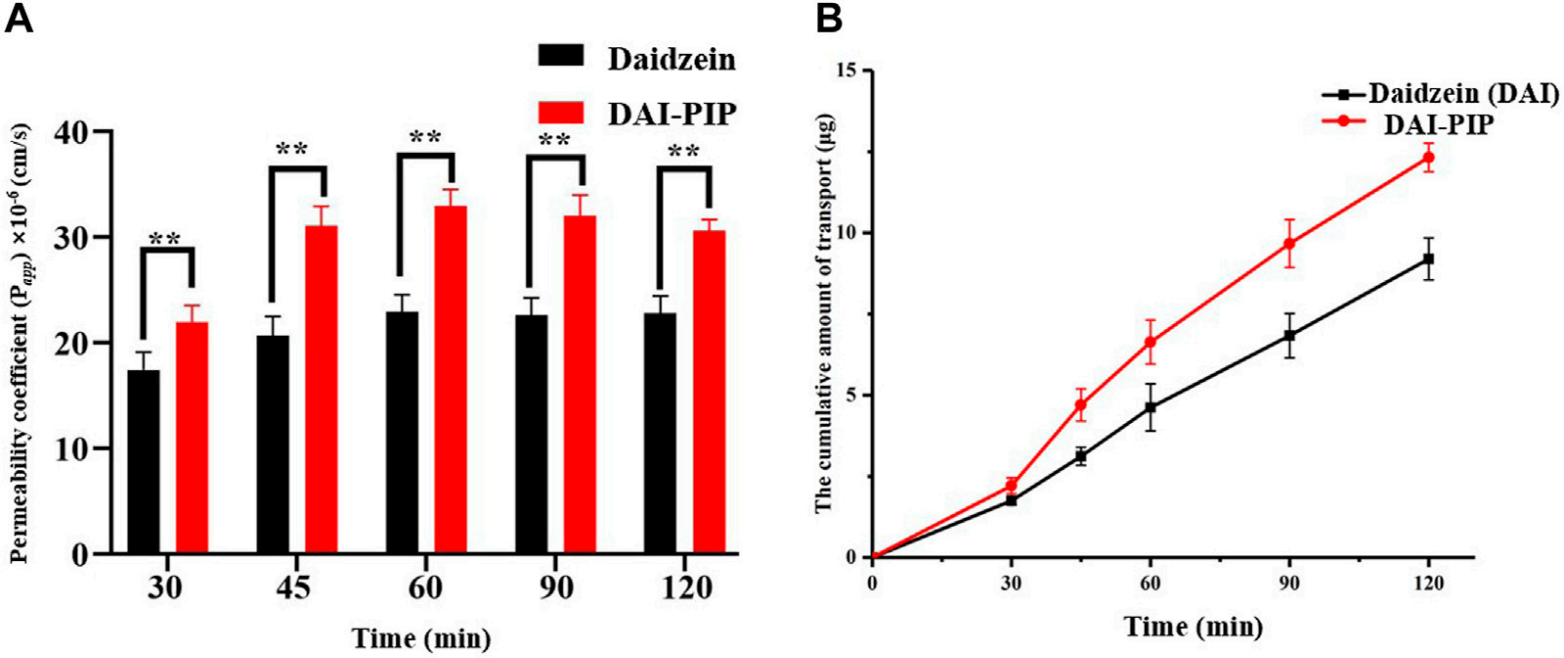
P_*app*_ value **(A)** and the cumulative amount **(B)** of transport of DAI and DAI from DAI-PIP.

DAI belongs to BCS class IV. In addition to dissolution performance, permeability plays an important role in determining the bioavailability of active ingredients (Kuche et al., 2019). The Caco-2 monolayer model has been well recognized for investigation of intestinal permeability of APIs (Zhu et al., 2013). It has been reported that DAI treatment increased claudin-1 expression, decreased tight junction (TJ) permeability, and enhanced the TEER of Caco-2 cell monolayers (Noda et al., 2012). Additionally, Kobayashi found that treating Caco-2 cells with BCRP inhibitor 3-sulfate estrone, P-gp inhibitor verapamil, and MRP inhibitor MK 571 increased the transport of DAI. This result indicates the recognition of DAI by efflux transporters and reflects its tendency to restrict intestinal absorption (Kobayashi et al., 2013). In this study, P_*app*_ values (from the AP side to BL side) and cumulative transport volume of pure DAI as well as DAI from DAI-PIP across Caco-2 cell monolayer were investigated. As shown in Figure 10, the P_*app*_ value of parent DAI was (22.80 ± 1.60) × 10^–6^ cm/s, which was consistent with an earlier report (Kawahara et al., 2020). However, the P_*app*_ value ((30.57 ± 1.08) ×10^–6^ cm/s) of DAI from DAI-PIP was 1.34-fold that of pure DAI. The cumulative transport increased gradually over time, and reached 9.19 ± 0.65 μg and 12.32 ± 0.43 μg (120 min) for pure DAI and DAI in DAI-PIP, respectively.

The higher permeability of DAI-PIP may be attributed to its elevated concentration in the solution, leading to the development of a concentration gradient of DAI and improvement in permeability. In addition, PIP might also play an important role, as it is known to enhance the permeation by modifying epithelial structure (Zheng et al., 2020). An investigation of 51 chemical permeation enhancers with diverse chemical structures revealed that two PIP derivatives achieved robust permeation enhancement in the Caco-2 model while inducing minimal cytotoxicity (Whitehead et al., 2008). Bzik and Brayden further confirmed the cell-permeation enhancing ability of PIP derivative and found that it exerts its effects by altering the expression of TJ proteins (Bzik and Brayden, 2016). Therefore, such improvements in both solubility and permeability may enhance the bioavailability of DAI.

### 4.8 *In Vivo* pharmacokinetics

**FIGURE 11:**
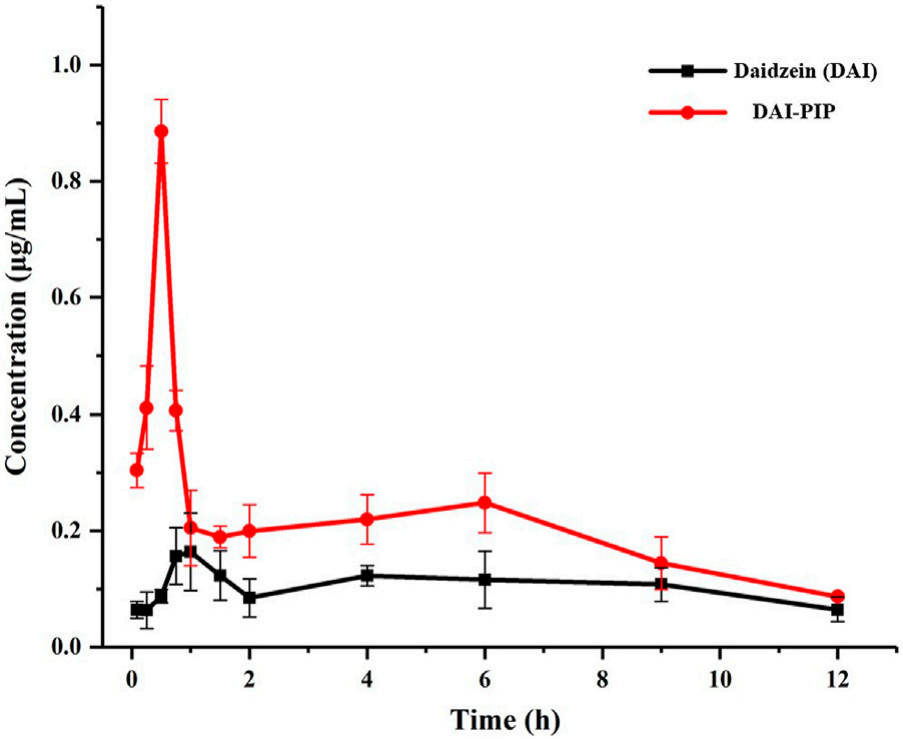
Plasma concentration–time profiles of DAI and DAI-PIP after oral administration.

The pharmacokinetic analysis of pure DAI and DAI-PIP was carried out in Beagle dogs. The plasma concentration-time profiles are shown in Figure 11. The calculated pharmacokinetic parameters including maximal plasma concentration (C_max_), time required to reach C_max_ (T_max_), and area under the curve (AUC) are presented in Table 5. For pure DAI, T_max_ was 0.83 ± 0.14 h with a C_max_ value of 0.21 ± 0.04 μg/mL, while the T_max_ value of DAI-PIP was 0.50 ± 0.14 h and C_max_ value was about 4.3-fold (0.91 ± 0.07 μg/mL) higher than that of pure DAI. This indicated that the new salt possessed faster absorption and higher C_max_. In addition, the AUC_0-24_ of DAI-PIP was about 2.4 times that of pure DAI. This enhanced bioavailability of the new salt may be attributed to its higher solubility, faster dissolution rate and greater permeability.

**TABLE 5:** Pharmacokinetic parameters of DAI and DAI-PIP.

Parameters	DAI	DAI-PIP
AUC_0∼24_ (μg·h/mL)	1.74 ± 0.15	4.15 ± 0.08**
C_max_ (μg/mL)	0.21 ± 0.04	0.91 ± 0.07**
T_max_ (h)	0.83 ± 0.14	0.5 ± 0.00

Note: Data in the same column without shoulder marks indicate insignificant difference (*p* > 0.05), * indicates significant difference (*p* < 0.05), ** indicates extremely significant difference (*p* < 0.01). (mean ± SD, n = 3).

## 5 Conclusion

DAI is classified as a BCS IV drug owing to its low solubility and permeability. In this study, a new salt of DAI using anhydrous PIP as a cocrystal former was successfully prepared and characterized. The new salt showed simultaneous enhancement in dissolution, permeability and oral bioavailability of DAI. Based on the above results, our future work will delve into the mechanism of action of DAI-PIP and evaluate its effectiveness in clinical applications.

## 6 Scope statement

We respectfully submit our manuscript entitled “A new crystalline Daidzein-piperazine salt with enhanced solubility, permeability and bioavailability” to be considered for publication in *Frontiers in Pharmacology*. In this study, a novel salt of daidzein (DAI) and piperazine (PIP) was prepared and comprehensively characterized in terms of its structure and physicochemical properties. Additionally, the permeability of DAI-PIP was assessed using the Caco-2 cell model and its pharmacokinetic behavior was explored in beagle dogs. The new salt showed simultaneous enhancement in dissolution, permeability and oral bioavailability of DAI.

The animal care and treatment followed the ethics guidelines set by the Animal Welfare Committee of Hebei Agricultural University Ethical Committee. Each author participated sufficiently in the study through conception, design, data analysis and interpretation, drafting and/or editing manuscript. All authors declare that there is no actual or potential conflict of interest with other persons and organizations. Thank you very much for your consideration of our work.

## Data Availability

The datasets presented in this study can be found in online repositories. The names of the repository/repositories and accession number(s) can be found in the article/Supplementary Material.
