# Supplementary figures and images for: A new crystalline daidzein-piperazine salt with enhanced solubility, permeability, and bioavailability
Source: Front Pharmacol. 2024 Jul 22;15:1385637. doi: 10.3389/fphar.2024.1385637 (PMC11298695; doi:10.3389/fphar.2024.1385637)

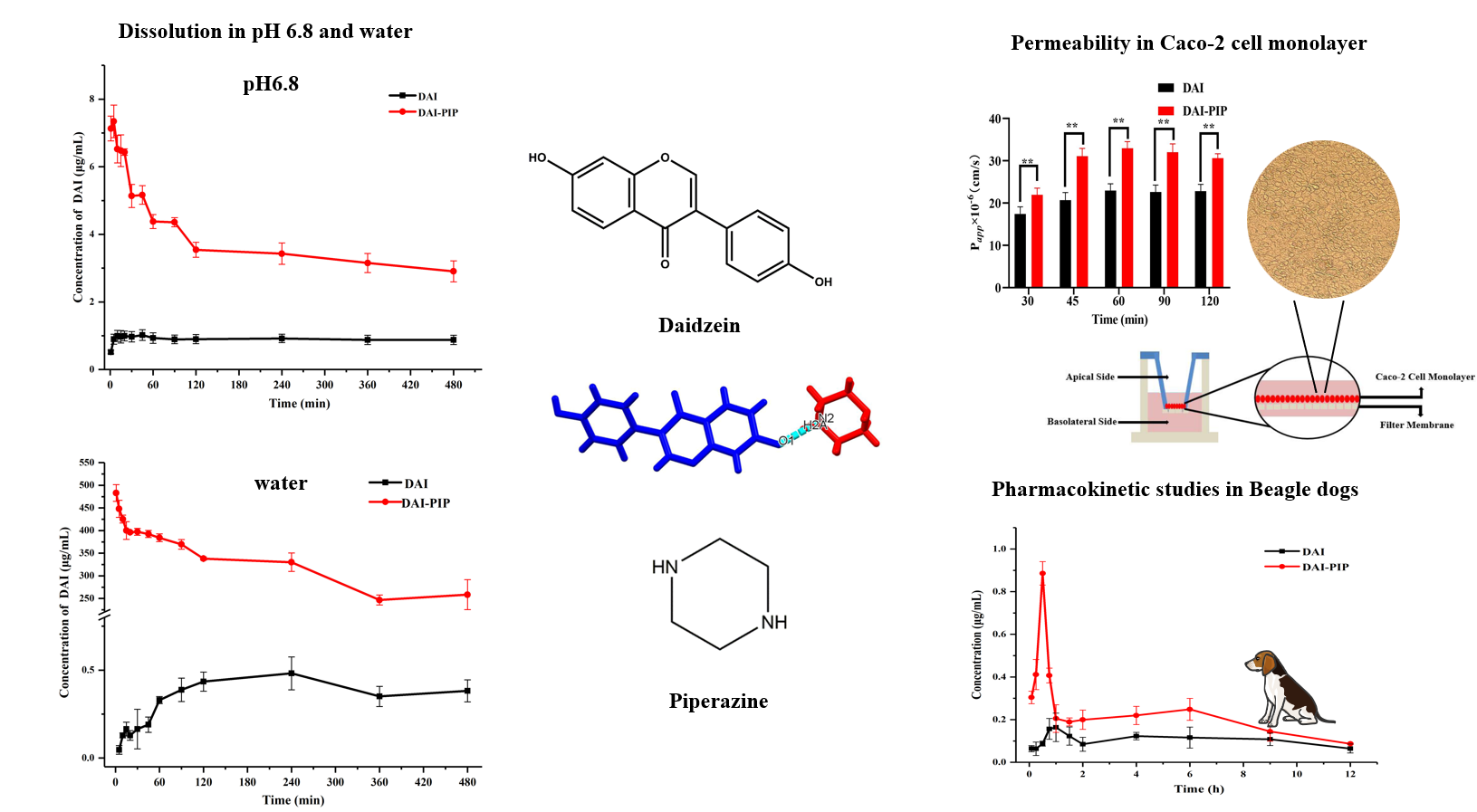

Supplement: Supplementary file 1 [file Image1.JPEG]
